# Prognostic value of total number of lymph nodes retrieved differs between left-sided colon cancer and right-sided colon cancer in stage III patients with colon cancer

**DOI:** 10.1186/s12885-018-4431-5

**Published:** 2018-05-11

**Authors:** Lin Yang, Zhenchong Xiong, Qiankun Xie, Wenzhuo He, Shousheng Liu, Pengfei Kong, Chang Jiang, Guifang Guo, Liangping Xia

**Affiliations:** 1 Sun Yat-sen University Cancer Center, 651 Dongfeng Road east, Guangzhou, 510060 China; 2 State Key Laboratory of Oncology in Southern China, Guangzhou, China; 3 Collaborative Innovation Center for Cancer Medicine, Guangzhou, China

**Keywords:** Total number of lymph nodes, Lymph node ratio), Left colon cancer, Right colon cancer, Overall survival (OS)

## Abstract

**Background:**

The consensus is that a minimum of 12 lymph nodes should be analyzed at colectomy for colon cancer. However, right colon cancer and left colon cancer have different characteristics, and this threshold value for total number of lymph nodes retrieved may not be universally applicable.

**Methods:**

The data of 63,243 patients with colon cancer treated between 2004 and 2012 were retrieved from the National Cancer Institute’s Surveillance, Epidemiology, and End Results database. Multivariate Cox regression analysis was used to determine the predictive value of total number of lymph nodes for survival after adjusting for lymph nodes ratio. The predictive value in left-sided colon cancer and right-sided colon cancer was compared. The optimal total number of lymph nodes cutoff value for prediction of overall survival was identified using the online tool Cutoff Finder. Survival of patients with high total number of lymph nodes (≥12) and low total number of lymph nodes (< 12) was compared by Kaplan–Meier analysis.

**Results:**

After stratifying by lymph nodes ratio status, total number of lymph nodes≥12 remained an independent predictor of survival in the whole cohort and in right-sided colon cancer, but not in left-sided colon cancer. The optimal cutoff value for total number of lymph nodes was determined to be 11. Low total number of lymph nodes (< 11) was associated with significantly poorer survival after adjusting for lymph nodes ratio in all subgroups except in the subgroup with high lymph nodes ratio (0.5–1.0).

**Conclusions:**

Previous reports of the prognostic significance of total number of lymph nodes on node-positive colon cancer were confounded by lymph nodes ratio. The 12-node standard for total number of lymph nodes may not be equally applicable in right-sided colon cancer and left-sided colon cancer.

**Electronic supplementary material:**

The online version of this article (10.1186/s12885-018-4431-5) contains supplementary material, which is available to authorized users.

## Background

In patients with colon cancer’ survival is independently associated with the number of lymph nodes analyzed at the time of colectomy [1]. This is true in both node-positive and node-negative disease, indicating that the benefit is not solely due to upstaging and administration of adjuvant therapy [1–3]. The American Joint Committee on Cancer recommends that a minimum of 7–14 lymph nodes be examined at colectomy to avoid understaging [4, 5]. The World Congress of Gastroenterology (1990) consensus was that at least 12 lymph nodes should be examined to ensure complete resection and adequate staging [1, 6, 7]. However, prognosis also depends on the status of regional lymph nodes, which is a major determinant of the need for adjuvant therapy.

Right-sided colon cancer and left-sided colon cancer have epidemiological, clinical, and molecular biological differences [8–10]. Right-sided colon cancer is more likely to belong to the consensus molecular subtype 2 (CMS2) and to show high frequency of DNA somatic copy number alterations (SCNA) and microsatellite stable/weak immune activation, which makes it relatively insensitive to immunotherapy [11]. It has even been suggested that right-sided colon cancer and left-sided colon cancer may be two different entities. Thus the recommendation that a minimum of 12 lymph nodesbe evaluated may not be equally applicable in right-sided colon cancer and left-sided colon cancer though, so far, there is little biologic evidence to support this theory.

The lymph node ratio—defined as number of positive lymph nodes /the number of total lymph nodes evaluated—independently predicts prognosis in stage III colon cancer [12] and may thus act as a confounder. Wang et al. found that the significance of the total number of lymph nodes as a quality-of-care measure in stage III colon cancer disappeared after adjusting for the effect of lymph nodes ratio [13]. However, they did not take tumor location into consideration. Additionally, their sample included patients treated from 1998 to 2003. Whether their conclusion remains valid in patients treated after 2003 is not known. Especially when a previous study has reported that between 1988 and 2011 there has been marked increase (from 20% to 80%) in the number of patients having ≥12 lymph nodes excised during colectomy for colon cancer [14].

Whether the lymph node ratio has the same confounding influence in both left-sided colon cancer and right-sided colon cancer remains unknown. The aim of this study was to evaluate whether the recommended standard of ≥12 for total number of lymph nodes has the same prognostic significance in right-sided colon cancer and left-sided colon cancer after adjusting for the lymph nodes ratio in a large national cohort.

## Methods

The data were obtained from the Surveillance, Epidemiology, and End Results (SEER) cancer registry. All patients receiving a first diagnosis of invasive stage III colon cancer (according to the 6th the American Joint Committee of cancer) criteria during the period from January 2004 through December 2012 were identified from the registry. The TNM stage was determined by SEER’s “extent of disease” (for T and M stage) and the “number of positive nodes” (for N stage) coding schemes. All patients had pathologically confirmed adenocarcinoma or mucinous adenocarcinoma [15], with tumor grade categorized as well differentiated, moderately differentiated, poorly differentiated, or undifferentiated. Colon cancer was evaluated as right-sided colon cancer (cecum, ascending colon, hepatic flexure, and transverse colon) or left-sided colon cancer (splenic flexure, descending colon, and sigmoid colon). The number of positive lymph node and the total number of lymph nodes evaluated were recorded, and lymph nodes ratio was calculated. Race/ethnicity was categorized as previously described. [16]

Patients were observed from 6 months after first diagnosis of colon cancer until the last follow-up, death, or end of the study, whichever occurred first.

### Statistical analysis

Patients were separated into four groups according to lymph nodes ratio value [12] as follows: lymph nodes ratio 1, < 0.07; lymph nodes ratio 2, 0.07–0.25, lymph nodes ratio 3, 0.25–0.50, and lymph nodes ratio 4, 0.50–1.0. Patients were also grouped according to total number of lymph nodes into high total number of lymph nodes (≥12) and low total number of lymph nodes (< 12) groups. Multivariate Cox proportional hazard model was used to evaluate the prognostic significance of total number of lymph nodes in left-sided colon cancer and right-sided colon cancer before and after adjustment for lymph nodes ratio. Kaplan–Meier analysis was used to estimate the survival difference between the different total number of lymph nodes and lymph nodes ratio subgroups. The optimal cutoff level for total number of lymph nodes was determined using the web-based application Cutoff Finder (http://medicine.yale.edu/lab/rimm/research/software.aspx) [17]. The data are presented in a triangular grid and each point represents a cut off value. The strength of the association of each cut-off point is reflected by the intensity of the color. We can move the cursor across the grid and the X-tile software allow the user to move a cursor across the grid and acquire the histogram of the resulting population subsets along with an associated Kaplan Meier curve. Then we can get the minimum *P* values from log-rank χ2 statistics for the total number of lymph nodes in terms of survival using the X-tile [18, 19].

Two-sided *P* < 0.05 was considered statistically significant. Statistical analysis was performed using SAS 9.2 (SAS Institute, Cary, NC, USA) and the survival package within R 2.11 (http://www.r-project.org).

## Results

### Patient characteristics

Totally, 63,243 stage III patients with colon cancer were identified in the SEER database. Among these patients, 39,024 could be classified as right-sided colon cancer and 24,219 as left-sided colon cancer. The present study included 30, 433 (48.1%)men and 32,810 (51.9%) women, ranging 19–82 years (median age: 69 years). 16,127 (25%) had < 12 lymph nodes excised (low total number of lymph nodes) and 47,116 (75%) had ≥12 lymph nodes excised (high total number of lymph). The proportion of high total number of lymph nodes patients was higher in right-sided colon cancer than in left-sided colon cancer (79% vs. 68%; Table 1).

**Table 1:** Clinical characteristics of patients in the full cohort, the Right-sided cohort, and the Left-sided cohort

Patients characteristics	Full cohort (*N* = 63,243		Right-sided (*N* = 39,024)		Left-sided (*N* = 24,219)	
	Total number of lymph nodes < 12	Total number of lymph nodes ≥12	Total number of lymph nodes < 12	Total number of lymph nodes ≥12	Total number of lymph nodes < 12	Total number of lymph nodes ≥12
	16,127 (25.5%)	47,116 (74.5%)	8370 (21.4%)	30,654 (78.6%)	7757 (32.0%)	16,462 (68.0%)
Age at diagnosis						
< 40	277 (1.7%)	1800 (3.8%)	93 (1.1%)	885 (2.9%)	184 (2.4%)	915 (5.6%)
40–49	872 (5.4%)	3631 (7.7%)	291 (3.5%)	1792 (5.8%)	581 (7.5%)	1839 (11.2%)
50–59	2473 (15.3%)	8658 (18.4%)	889 (10.6%)	4658 (15.2%)	1584 (20.4%)	4000 (24.3%)
60–69	3664 (22.7%)	10,934 (23.2%)	1719 (20.5%)	6984 (22.8%)	1945 (25.1%)	3950 (24.0%)
> 70	8841 (54.8%)	22,093 (46.9%)	5378 (64.3%)	16,335 (53.3%)	3463 (44.6%)	5758 (35.0%)
Gender						
Male	8011 (49.7%)	22,422 (47.6%)	3828 (45.7%)	13,797 (45.0%)	4183 (53.9%)	8625 (52.4%)
Female	8116 (47.6%)	24,694 (52.4%)	4542 (54.3%)	16,857 (55.0%)	3574 (46.1%)	7837 (47.6%)
Race						
Black	2144 (13.3%)	5796 (12.3%)	1113 (13.3%)	3805 (12.4%)	1031 (34.1%)	1991 (12.1%)
White	12,446 (77.2%)	37,081 (78.7%)	6691 (79.9%)	24,651 (80.4%)	5755 (74.2%)	12,430 (75.5%)
Hispanic/Latino	119 (0.7%)	296 (0.6%)	69 (0.8%)	181 (0.6%)	50 (0.6%)	115 (0.7%)
Asian or Pacific Islander and others	1378 (8.5%)	3784 (8.0%)	484 (5.8%)	1922 (6.3%)	894 (11.5%)	1862 (11.3%)
Unknown	40 (0.2%)	159 (0.3%)	13 (0.2%)	95 (0.3%)	27 (0.3%)	64 (0.4%)
Hispanic						
No	14,460 (89.7%)	42,480 (90.2%)	7579 (90.5%)	27,800 (90.7%)	6881 (88.7%)	14,680 (89.2%)
Yes	1667 (10.3%)	4636 (9.8%)	791 (9.5%)	2854 (9.3%)	876 (11.3%)	1782 (10.8%)
T stage						
T0–T2 (0, 2, 3)	2723 (16.9%)	5553 (11.8%)	1144 (13.7%)	3437 (11.2%)	1579 (20.4%)	2116 (12.9%)
T3–T4 (4, 5)	13,376 (82.9%)	41,503 (88.1%)	7216 (86.2%)	27,181 (88.7%)	6160 (79.4%)	14,322 (87.0%)
TX (6)	28 (0.2%)	60 (0.1%)	10 (0.1%)	36 (0.1%)	18 (0.2%)	24 (0.1%)
N stage						
N1	12,005 (74.4%)	29,678 (63.0%)	6057 (72.4%)	19,100 (62.3%)	5948 (76.7%)	1809 (23.3%)
N2	4122 (25.6%)	17,438 (37.0%)	2313 (27.6%)	11,554 (37.7%)	10,578 (64.3%)	5884 (35.7%)
Chemotherapy						
No	8200 (50.8%)	19,558 (41.5%)	4640 (55.4%)	13,549 (44.6%)	3560 (45.9%)	6009 (36.5%)
Yes	7927 (49.2%)	27,558 (58.5%)	3730 (44.6%)	17,105 (55.8%)	4197 (54.1%)	10,453 (63.5%)
Radiotherapy						
No	37 (0.2%)	88 (0.2%)	18 (0.2%)	57 (0.2%)	19 (0.2%)	31 (0.2%)
Yes	343 (2.1%)	868 (1.8%)	107 (1.3%)	298 (1.0%)	236 (3.0%)	570 (3.5%)
Unknown	15,747 (97.6%)	46,160 (98.0%)	8245 (98.5%)	30,299 (98.8%)	7502 (96.7%)	15,861 (96.3%)
Marital status						
Single, separated, divorced,	3574 (22.2%)	11,237 (23.8%)	1734 (20.7%)	6932 (22.6%)	1840 (23.7%)	4305 (26.2%)
Married (including common law)	8568 (53.1%)	25,176 (53.4%)	4223 (50.5%)	15,937 (52.0%)	4345 (56.0%)	9239 (56.1%)
Widowed	3362 (20.8%)	8714 (18.5%)	2053 (24.5%)	6469 (21.1%)	1309 (16.9%)	2245 (13.6%)
Unknown	623 (3.9%)	1989 (4.2%)	360 (4.3%)	1316 (4.3%)	263 (3.4%)	673 (4.1%)
Pathology grade						
Well-differentiated	999 (6.2%)	2440 (5.2%)	483 (5.8%)	1539 (5.0%)	516 (6.7%)	901 (5.5%)
Moderately differentiated	10.677 (66.2%)	29,917 (63.5%)	5042 (60.2%)	18,005 (58.7%)	5635 (72.6%)	11,912 (72.4%)
Poorly differentiated	3619 (22.4%)	11,956 (25.4%)	2338 (27.9%)	8995 (29.3%)	1281 (16.5%)	2961 (18.0%)
Undifferentiated	401 (2.5%)	1802 (3.8%)	274 (3.3%)	1431 (4.7%)	127 (1.6%)	371 (2.3%)
Unknown	431 (2.7%)	1.001 (2.1%)	233 (2.8%)	684 (2.2%)	198 (2.6%)	317 (1.9%)
Positive lymph node						
0	421 (2.6%)	971 (2.1%)	193 (2.3%)	576 (1.9%)	228 (2.9%)	395 (2.4%)
1	6032 (37.4%)	14,198 (30.1%)	3050 (36.4%)	9165 (29.9%)	2982 (38.4%)	5033 (30.6%)
2–3	5483 (34.0%)	14,244 (30.2%)	2776 (33.2%)	9176 (29.9%)	2707 (34.9%)	5068 (30.8%)
4–6	3023 (18.7%)	9226 (19.6%)	1621 (19.4%)	6008 (19.6%)	1402 (18.1%)	3218 (19.5%)
≥ 7	1168 (7.2%)	8477 (18.0%)	730 (8.7%)	5729 (18.7%)	438 (5.6%)	2748 (16.7%)
Lymph nodes ratio						
< 0.07	421 (2.6%)	14,484 (30.7%)	193 (2.3%)	9518 (31.0%)	228 (2.9%)	4966 (30.2%)
0.07–0.25	7089 (44.0%)	20,189 (42.8%)	3673 (43.9%)	12,965 (42.3%)	3416 (44.0%)	7224 (43.9%)
0.25–0.50	4645 (28.8%)	7954 (16.9%)	2361 (28.2%)	5172 (16.9%)	2284 (29.4%)	2782 (16.9%)
0.50–1.0	3972 (24.6%)	4489 (9.5%)	2143 (25.6%)	2999 (9.8%)	1829 (23.6%)	1490 (9.1%)
Tumor site						
Right-sided	8370 (51.9%)	30,654 (65.1%)				
Left-sided	7757 (48.1%)	16,462 (34.9%)				

The total number of lymph nodes ranged from 1 to 90 in the entire cohort, with a median of 17. Median total number of lymph nodes was 18 and 15, respectively, in right-sided colon cancer and left-sided colon cancer. The proportion of patients with 1, 2–3, 4–6, or ≥ 7 positive nodes was comparable in the high total number of lymph nodes and low total number of lymph nodes groups. In addition, the proportion of patients with definitely positive lymph nodes was similar in left-sided colon cancer and right-sided colon cancer.

Total number of lymph nodes < 12 and ≥ 12 were seen in 3% vs. 31% patients in lymph nodes ratio1; 44% vs. 43% patients in lymph nodes ratio 2; 29% vs. 17% patients in lymph nodes ratio 2; and 25% vs. 10% patients in lymph nodes ratio 4. The difference between the proportions of low total number of lymph nodes and high total number of lymph nodes was significant in lymph nodes ratio 1, lymph nodes ratio 3, and lymph nodes ratio 4. Correspondingly, there were significant difference between the proportions of lymph nodes ratio1, lymph nodes ratio 3, and lymph nodes ratio 4 in the high total number of lymph nodes and low total number of lymph nodes groups in both right-sided colon cancer and left-sided Patients with colon cancer. Table 1 shows the likelihood of having the recommended 12 lymph nodes excised, classified by clinical feature and demographic characteristic.

### Univariate and multivariate cox regression analysis

The univariate Cox proportional hazard model showed that high total number of lymph nodes patients had a 24% lower probability of death than low total number of lymph nodes patients patients (HR = 0.758, 95% CI, 0.739–0.778; *P* < 0.001) (Table 2). In multivariate Cox regression analysis high total number of lymph nodes patients patients had 25% lower probability of death (HR = 0.747, 95% CI, 0.728–0.726; *P* < 0.001) when no adjustment was made for lymph nodes ratio (Table 3). Interestingly, however, after adjusting for lymph nodes ratio, high total number of lymph nodes patients had a 6% higher probability of death than low total number of lymph nodes patients (HR = 0.938, 95% CI, 0.909–0.967; *P* < 0.001).

**Table 2:** Univariate Cox regression analysis of the whole cohort, the Right-sided cohort, and the Left-sided cohort

Patients Characteristics	Full cohort		Right-sided		Left-sided	
	HR (95% CI)	P	HR (95% CI)	P	HR (95% CI)	P
Age at diagnosis		< 0.001		< 0.001		< 0.001
< 40	1.000		1.000		1.000	
40–49	1.101 (0.987–1.229)	0.319	1.080 (0.928–1.258)	0.319	1.126 (0.961–1.320)	0.143
50–59	1.158 (1.048–1.279)	0.007	1.205 (1.051–1.381)	0.007	1.086 (0.938–1.256)	0.270
60–69	1.557 (1.414–1.716)	< 0.001	1.499 (1.313–1.711)	< 0.001	1.523 (1.321–1.757)	< 0.001
> 70	3.364 (3.063–3.695)	< 0.001	2.999 (2.636–3.411)	< 0.001	3.646 (3.178–4.183)	< 0.001
Gender						
Female vs. Male	0.989 (0.960–1.019)	0.018	0.475	0.875	0.839–0.913	< 0.001
Race		< 0.001		< 0.001		< 0.001
Black	1.000		1.000		1.000	
White	0.994 (0.959–1.031)	0.756	1.056 (1.010–1.105)	0.017	0.879 (0.827–0.935)	< 0.001
Hispanic/Latino	0.861 (0.730–1.016)	0.077	0.913 (0.743–1.122)	0/388	0.793 (0.601–1.046)	0.101
Asian or Pacific Islander and others	0.699 (0.659–0.742)	< 0.001	0.766 (0.708–0.828)	< 0.001	0.671 (0.614–0.734)	< 0.001
Unknown	0.445 (0.320–0.618)	< 0.001	0.449 (0.295–0.684)	< 0.001	0.449 (0.265–0.761)	0.003
Hispanic						
Yes vs. No	0.834 (0.799–0.871)	< 0.001	0.838 (0.793–0.885)	< 0.001	0.857 (0.798–0.921)	< 0.001
T stage		< 0.001		< 0.001		< 0.001
T0–T2 (0, 2, 3)	1.000		1.000		1.000	
T3–T4 (4, 5)	1.973 (1.889–2.061)	< 0.001	1.784 (1.690–1.883)	< 0.001	2.212	2.055–2.381
TX (6)	1.315 (0.900–1.922)	0.157	1.188 (0.714–1.976)	0.507	1.575 (0.891–2.786)	0.118
N stage						
N2 vs. N1	1.581 (1.543–1.620)	< 0.001	1.657 (1.608–1.707)	< 0.001	1.415 (1.355–1.477)	< 0.001
Chemotherapy						
Yes vs. No	0.408 (0.398–0.418)	< 0.001	0.430 (0.417–0.443)	< 0.001	0.386 (0.370–0.403)	< 0.001
Radiotherapy		< 0.001		< 0.001		0.001
No	1.000		1.000		1.000	
Yes	0.517 (0.410–0.654)	< 0.001	0.637 (0.469–0.865)	0.004	0.508 (0.351–0.735)	< 0.001
Unknown	0.524 (0.421–0.651)	< 0.001	0.515 (0.390–0.680)	< 0.001	0.506 (0.356–0.721)	< 0.001
Marital status		< 0.001		< 0.001		< 0.001
Single, separated, divorced,	1.000		1.000		1.000	
Married (including common law)	0.843 (0.818–0.870)	< 0.001	0.872 (0.839–0.907)	< 0.001	0.788 (0.748–0.829)	< 0.001
Widowed	1.681 (1.623–1.740)	< 0.001	1.592 (1.524–1.659)	< 0.001	1.746 (1.643–1.856)	< 0.001
Unknown	1.065 (0.996–1.139)	0.065	1.082 (0.998–1.173)	0.056	0.984 (0.873–1.110)	0.792
Pathology grade		< 0.001		< 0.001		< 0.001
Well-differentiated	1.000		1.000		1.000	
Moderately differentiated	1.119 (1.056–1.186)	< 0.001	1.197 (1.112–1.289)	< 0.001	1.020 (0.930–1.119)	0.669
Poorly differentiated	1.635 (1.540–1.736)	< 0.001	1.673 (1.551–1.804)	< 0.001	1.436 (1.300–1.586)	< 0.001
Undifferentiated	1.981 (1.825–2.150)	< 0.001	1.988 (1.802–2.192)	< 0.001	1.735 (1.476–2.040)	< 0.001
Unknown	1.308 (1.188–1.439)	< 0.001	1.403 (1.247–1.578)	< 0.001	1.113 (0.942–1.315)	0.208
Positive lymph node		< 0.001		< 0.001		< 0.001
0	1.000		1.000		1.000	
1	0.943 (0.851–1.045)	0.265	0.933 (0.819–1.062)	0.293	0.932 (0.787–1.105)	0.418
2–3	1.085 (0.979–1.202)	0.121	1.082 (0.951–1.232)	0.232	1.058 (0.893–1.253)	0.514
4–6	1.347 (1.215–1.495)	< 0.001	1.385 (1.216–1.578)	< 0.001	1.236 (1.041–1.467)	0.015
≥ 7	2.012 (1.814–2.232)	< 0.001	2.101 (1.844–2.393)	< 0.001	1.713 (1.441–2.035)	< 0.001
Lymph nodes ratio		< 0.001		< 0.001		< 0.001
< 0.07	1.000		1.000		1.000	
0.07–0.25	1.312 (1.267–1.360)	< 0.001	1.375 (1.318–1.435)	< 0.001	1.245 (1.168–1.327)	< 0.001
0.25–0.50	1.771 (1.704–1.842)	< 0.001	1.942 (1.853–2.036)	< 0.001	1.597 (1.490–1.711)	< 0.001
0.50–1.0	2.873 (2.761–2.990)	< 0.001	3.302 (3.147–3.466)	< 0.001	2.446 (2.279–2.625)	< 0.001
Tumor site						
Left-sided vs. Right-sided	0.716 (0.698–0.734)	< 0.001				
^a^Total number of lymph node						
≥ 12 vs. < 12	0.758 (0.739–0.778)	< 0.001	0.692 (0.670–0.715)	< 0.001	0.765 (0.733–0.798)	< 0.001
^b^Total number of lymph node	0.985 (0.983–0.986)	< 0.001	0.980 (0.978–0.982)	< 0.001	0.986 (0.983–0.988)	< 0.001
^c^Total number of lymph nodes						
≥ 11 vs. < 11					0.742 (0.706–0.779)	< 0.001

Abbreviations: ^a^Total number of lymph nodes as the Categorical variable

^b^Total number of lymph nodes as a continuous variable

^c^The threshold of total number of lymph nodes as 11 in the Left-sided cohort

**Table 3:** Multivariate Cox Regression for the whole cohort, the Right-sided cohort and the Left-sided cohort

Patients Characteristics	Full cohort		Right-sided		Left-sided	
	HR (95% CI)	P	HR (95% CI)	P	HR (95% CI)	P
Age at diagnosis		< 0.001		< 0.001		< 0.001
< 40	1.000		1.000		1.000	
40–49	1.074 (0.962–1.199)	0.201	1.019 (0.875–1.187)	0.809	1.146 (0.977–1.343)	0.094
50–59	1.144 (1.035–1.264)	.0.008	1.162 (1.014–1.333)	0.031	1.107 (0.956–1.281)	0.174
60–69	1.466 (1.330–1.616)	< 0.001	1.422 (1.245–1.624)	< 0.001	1.462 (1.266–1.688)	< 0.001
> 70	2.516 (2.287–2.769)	< 0.001	2.265 (1.987–2.581)	< 0.001	2.878 (2.500–3.312)	< 0.001
Sex						
Female vs. Male	0.804 (0.784–0.825)	< 0.001			0.796 (0.762–0.833)	< 0.001
Race		< 0.001		< 0.001		< 0.001
Black	1.000		1.000		1.000	
White	0.866 (0.834–0.898)	< 0.001	0.907 (0.866–0.950)	< 0.001	0.797 (0.749–0.848)	< 0.001
Hispanic/Latino	0.848 (0.718–1.001)	0.051	0.856 (0.696–1.052)	0.140	0.825 (0.625–1.090)	0.175
Asian or Pacific Islander and others	0.687 (0.647–0.729)	< 0.001	0.699 (0.646–0.757)	< 0.001	0.663 (0.606–0.726)	< 0.001
Unknown	0.471 (0.339–0.654)	< 0.001	0.482 (0.317–0.734)	0.001	0.462 (0.273–0.783)	0.004
Hispanic						
Yes vs. No	0.920 (0.881–0.962)	< 0.001	0.906 (0.857–0.958)	< 0.001		
T stage		< 0.001				< 0.001
T0-T2 (0, 2, 3)	1.000		1.00		1.000	
T3-T4 (4, 5)	1.703 (1.629–1.780)	< 0.001	1.540 (1.458–1.627)	< 0.001	1.984 (1.841–2.138)	< 0.001
TX (6)	1.366 (0.933–1.999)	0.109	1.155 (0.693–1.926)	0.580	1.756 (0.991–3.112)	0.054
N stage						
N2 vs. N1	1.139 (1.097–1.181)	< 0.001	1.157 (1.105–1.211)	< 0.001	1.063 (1.005–1.124)	0.033
Chemotherapy						
Yes vs. No	0.499 (0.486–0.513)	< 0.001	0.501 (0.485–0.517)	< 0.001	0.491 (0.469–0.513)	< 0.001
Radiotherapy				< 0.001		
No	1.000		1.000		1.000	
Yes	1.286 (1.017–1.626)	0.036	1.402 (1.031–1.906)	0.031	1.253 (0.865–1.816)	0.233
Unknown	0.866 (0.696–1.077)	0.196	0.851 (0.644–1.123)	0.254	0.886 (0.623–1.262)	0.503
Marital status		< 0.001		< 0.001		< 0.001
Single, separated, divorced,	1.000		1.000		1.000	
Married (including common law)	0.785 (0.761–0.811)	< 0.001	0.844 (0.811–0.878)	< 0.001	0.733 (0.695–0.773)	< 0.001
Windowed	1.078 (1.039–1.120)	< 0.001	1.051 (1.005–1.099)	< 0.001	1.029 (0.963–1.099)	0.399
Unknown	0.875 (0.818–0.936)	< 0.001	0.904 (0.834–0.981)	0.015	0.812 (0.720–0.916)	0.001
Pathology grade		< 0.001		< 0.001		< 0.001
Well-differentiated	1.000		1.000		1.000	
Moderately differentiated	1.086 (1.025–1.151)	0.005	1.145 (1.063–1.233)	< 0.001	1.003 (0.915–1.101)	0.942
Poorly differentiated	1.340 (1.262–1.424)	< 0.001	1.378 (1.276–1.487)	< 0.001	1.282 (1.160–1.417)	< 0.001
Undifferentiated	1.636 (1.507–1.777)	< 0.001	1.658 (1.502–1.830)	< 0.001	1.639 (1.393–1.928)	< 0.001
Unknown	1.228 (1.115–1.352)	< 0.001	1.275 (1.133–1.435)	< 0.001	1.157 (0.979–1.368)	0.087
Tumor site						
Left-sided vs. Right-sided	0.847 (0.824–0.870)	< 0.001				
Lymph nodes ratio		< 0.001		< 0.001		< 0.001
< 0.07	1.000		1.000		1.000	
0.07–0.25	1.277 (1.229–1.325)	< 0.001	1.294 (1.236–1.354)	< 0.001	1.245 (1.167–1.328)	< 0.001
0.25–0.50	1.602 (1.523–1.685)	< 0.001	1.663 (1.563–1.770)	< 0.001	1.514 (1.402–1.636)	< 0.001
0.50–1.0	2.494 (2.357–2.640)	< 0.001	2.671 (2.491–2.863)	< 0.001	2.282 (2.100–2.480)	< 0.001
^a^Total number of lymph nodes (without Lymph nodes ratio)						
≥ 12 vs. < 12	0.747 (0.728–0.766)	< 0.001	0.706 (0.683–0.730)	< 0.001	0.780 (0.747–0.815)	< 0.001
Total number of lymph nodes (with Lymph nodes ratio)						
≥ 12 vs. < 12	0.938 (0.909–0.967)	< 0.001	0.902 (0.886–0.957)	< 0.001	0.968 (0.919–1.020)	0.223
^b^Total number of lymph nodes (without Lymph nodes ratio)	1.000 (0.998–1.001)	0.694	0.981 (0.980–0.983)	< 0.001	0.985 (0.982–0.987)	< 0.001
Total number of lymph nodes (with Lymph nodes ratio)	1.004 (1.003–1.006)	< 0.001	0.994 (0.992–0.997)	< 0.001	0.996 (0.993–1.000)	0.033
^c^Total number of lymph nodes (without Lymph nodes ratio)						
≥ 11 vs. < 11					0.704 (0.669–0.740)	< 0.001
Total number of lymph nodes (with Lymph nodes ratio)						
≥ 11 vs. < 11					0.868 (0.813–0.927)	< 0.001

Abbreviations: ^a^Total number of lymph nodes as a Categorical variable

^b^Total number of lymph nodes as a continuous variable

^c^The threshold is 11 in the Left-sided cohort

In univariate analysis, the probability of death was lower in high total number of lymph nodes patients than in low total number of lymph nodes patients in both right-seided colon cancer (lower by 31%) and left-sided colon cancer (lower by 23%) (Table 2). In multivariate Cox regression without adjusting for lymph nodes ratio, these figures were still relatively high (29% and 22% in right-sided colon cancer and left-sided colon cancer, respectively) (Table 3). After adjusting for lymph nodes ratio, the survival advantage of high total number of lymph nodes disappeared in left-sided colon cancer but persisted in right-sided colon cancer where, however, the survival advantage was now only 3% higher for high total number of lymph nodes patients.

To avoid the influence of using the empirical cutoff points for the stratification of lymph nodes ratio and total number of lymph nodes, the previous analysis was repeated by utilizing using both lymph nodes ratio and total number of lymph nodes both as continuous variables. In the univariate Cox regression, for each unit increase in total number of lymph nodes the probability of death decreased by 1%, 1%, and 1%, respectively, in the whole cohort, the right-sided colon cancer cohort, and the left-sided colon cancer cohort (Table 2). In the univariate Cox mode, the lower chance of death for each additional total number of lymph nodes in a patient were 1%, 1% and 1% for the whole cohort, the right-sided colon cancer cohort and the left-sided colon cancer cohort (HR = 0.985, 95% CI = 0.983–0.986, *P* < 0.001; HR = 0.90, 0.978–0.982, *P* < 0.001; HR = 0.986, 95% CI, 0.983–0.988, *P* < 0.001; respectively) (Table 3). Paradoxically, after the adjustment for lymph nodes ratio, a patient has 0.4% higher chance probability of death for each additional unit increase in total number of lymph nodes (HR = 1.004, 95% CI, 1.003–1.006; *P* < 0.0001) (Table 3). In multivariate analysis, total number of lymph nodes was a predictor of survival, irrespective of whether or not adjustment was made for lymph nodes ratio.

### Optimal cutoff for total number of lymph nodes in left-sided colon cancer

**Fig. 1:**
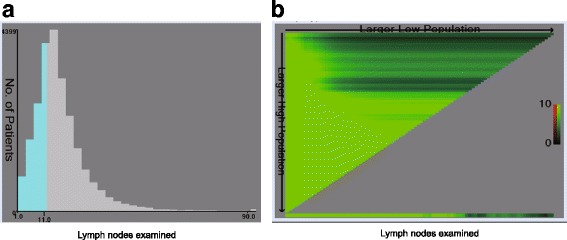
**a** The number of lymph nodes distribution according to lymph nodes examined. Ranged from 0 to 90. **b** X-tile plots for lymph nodes constructed by left-sided Patients with colon cancer. The plots dividing them into 2 groups by the cutoff point 10. The brightest pixel represents the maximum χ2 log-rank value

Using the Cutoff Finder tool, we determined the optimal cutoff for total number of lymph nodes in left-sided colon cancer to be 11; this value provided the greatest separation of the OS curves in Kaplan–Meier analysis (Fig. 1). Univariate analysis showed that in the left-sided colon cancer cohort, patients with total number of lymph nodes ≥11 had 26% greater probability of survival (HR = 0.742, 95% CI, 0.706–0.779; *P* < 0.001) than patients with total number of lymph nodes < 11. In the multivariate Cox regression, the higher probability of survival persisted, irrespective of whether or not adjustment was made for lymph nodes ratio (HR = 0.704, 95% CI, 0.669–0.740; *P* < 0.001; without adjustment for lymph nodes ratio and HR = 0.868, 95% CI, 0.813–0.927; *P* < 0.001; after adjustment for lymph nodes ratio).

### Survival comparison

**Fig. 2:**
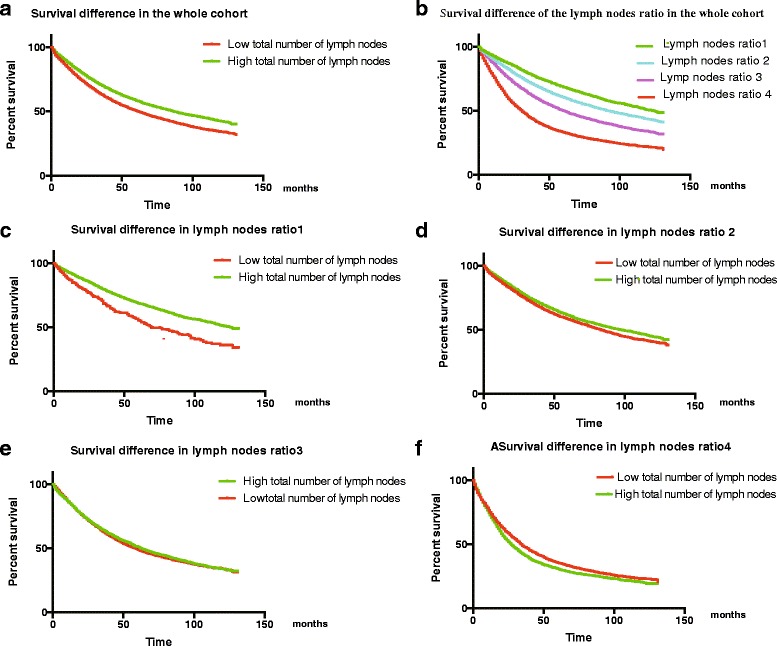
**a** Survival curves of patients with low and high total number of lymph nodes. **b** Survival curves stratified by lymph nodes ratio. **c** Survival of lymph nodes ratio 1 patients according to total number of lymph nodes. **d** Survival of lymph nodes ratio 2 patients according to total number of lymph nodes. **e** Survival of lymph nodes ratio 3 patients according to total number of lymph nodes. **f** Survival of lymph nodes ratio 4 patients according to total number of lymph nodes

The 5-year survival rate was 59% in the high total number of lymph nodes subgroup, which was 1.2 times higher than that in the low total number of lymph nodes subgroup (Fig. 2a). Survival was better in high total number of lymph nodes patients in both right-sided colon cancer and left-sided colon cancer (Additional file 1: Figure S1A and Additional file 2: Figure S2A for right-sided colon cancer and left-sided colon cancer, respectively). The 5-year survival in each lymph nodes ratio subgroup was as follows: 65% in lymph nodes ratio 1, 56% in lymph nodes ratio 2, 45% in lymph nodes ratio 3, and 30% in lymph nodes ratio 4; the difference between the groups was statistically significant (*P* < 0.001; Fig. 2b). The survival differences between the lymph nodes ratio strata were statistically significant in both right-sided colon cancer and left-sided colon cancer (*P* < 0.0001; Additional file 1: Figure S1B and Additional file 2: Figure S2B for right-sided colon cancer and left-sided colon cancer, respectively).

An unexpected finding from Kaplan–Meier survival analysis was that high total number of lymph nodes was associated with significantly better survival in the lymph nodes ratio < 0.25 subgroup but not in the higher lymph nodes ratio subgroups (Fig. 2c–f,low total number od lymph nodes vs high total number od lymph nodes, HR = 1.257, 95% CI, 1.165–1.357, *P* < 0.001, for Fig. 2c; HR = 1.074, 95% CI, 1.052–1.096, =*P* < 0.001, for Fig. 2d; HR = 1.006,95% CI 0.981–1.033, *P* = 0.619, for Fig. 2e; HR = 0.956,95% CI, 0.931–0.982, *P* < 0.001, for Fig. 2f). When assessed in the context of the right-sided colon cancer cohort, however, this difference in mortality also merely appears to be exists in the lymph nodes ratio < 0.25 and the high total number of lymph nodes have better survival than the low total number of lymph nodes in the left-sided colon cancer cohort of the strata lymph nodes ratio < 0.5 with the threshold of lymph nodes of 10 (Additional file 1: Figure S1C–F and Additional file 2: Figure S2C–F for right-sided colon cancer and left-sided colon cancer, respectively).

## Discussion

The relationship between the total number of lymph nodes and outcome of colon cancer has been extensively studied. [1, 20–27] Our finding of improved survival with higher lymph node yield is consistent with previous research [1, 20–23, 25, 27–31]. The mechanism remains unknown, but potential factors include more accurate tumor staging, improved surgical management, and superior quality of pathology service [32]. Some studies have demonstrated that a stronger host immune response [33] and the molecular/biological characteristics of the tumor are related to high total number of lymph nodes, and these too may be responsible for its effect on prognosis [32, 34]. For a long time, the presumed reason was that more extensive lymph node evaluation leads to less understaging, and thereby to better treatment and survival. However, LeVoyer et al. [1] and Prandi et al. [35] have all found that increase in the number of lymph nodes retrieved did not necessarily result in upstaging or in increased survival. [36] These authors also showed that the node positivity rate did not change despite a steep increase in total number of lymph nodes, and that it is important to consider the effect of node positivity rate on prognosis. Wong et al. [37] found that distribution of the positive lymph node frequency and the rate of receiving adjuvant chemotherapy are similar among hospital groups although the number of lymph nodes evaluated varied widely, suggesting that increasing total number of lymph nodes does not significantly upstage patients.

In our study, we found all lymph nodes ratio strata were independently predictive of overall survival. Thus, although the 12-node-minimum standard remains an important prognostic determinant, lymph nodes ratio has a separate contributory prognostic role even though the surgical benchmark and lymph nodes ratio are interrelated parameters. Importantly, our study showed no relationship between total number of lymph nodes and outcome in the left-sided colon cancer cohort when adjustment was made for lymph nodes ratio in multivariate analysis; this finding is consistent with Wang et al. [13]

The American Joint Committee on cancer TNM staging system is based on number of positive nodes, not lymph nodes ratio. In the present study, Kaplan–Meier survival analysis showed that high total number of lymph nodes is associated with significantly improved survival in the lymph nodes ratio < 0.25 subgroup. However, the difference between survival of high total number of lymph nodes and low total number of lymph nodes patients was less in the higher lymph nodes ratio strata. Furthermore, the distribution of lymph nodes ratio strata between the high total number of lymph nodes and low total number of lymph nodes groups was significantly different. This may because low lymph nodes ratio subgroups have lower metastasis risk and fewer positive lymph nodes, and so most of the lymph nodes retrieved during surgery are negative lymph nodes. The fewer number of positive nodes retrieved may cause an artificial inflation of the lymph nodes ratio—the “small-denominator effect.” Conversely, the high lymph nodes ratio subgroups have relatively higher metastasis risk and more number of positive lymph nodes. Although more lymph nodes are retrieved in the high total number of lymph nodes, the number of positive lymph nodes is also high, and the tumor burden is the leading role not the immune reaction. In this occasion, a “large-denominator” effect may be an issue as it is much easier to achieve positive nodes sampled.

A threshold total number of lymph nodes of 12 is often used as a measure of quality of surgical care. However, it may not be adequate to only use the number of lymph nodes evaluated as a quality-of-care measure; the surgeon’s technique and the pathologist’s methods must also be taken into account. Other variables, such as the patient’s immunologic response and age [38] and the location of the primary tumor, may also greatly impact the number of lymph nodes retrieved. Prandi et al. have shown that the number of lymph nodes retrieved is inversely associated with patient’s age [35]. Another study found that more lymph nodes are identified in patients with right-sided colon cancer, which led the authors to question whether the standard for regional lymph nodes evaluation should be the same in right-sided colon cancer and left-sided colon cancer [39].

Our study showed that after adjusting for the lymph nodes ratio, the association of total number of lymph nodes with survival in stage IIII colon cancer was present only in right-sided colon cancer but not in left-sided colon cancer. The biological behavior of the tumor and host parameters such as immune response may affect the number of traceable lymph nodes. Several authors have hypothesized that smaller numbers of lymph nodes found is reflection of a diminished immune response. Size and morphology of nodes are modified by immune responses, with a weak immune response leading to smaller nodes that are harder to find [21, 25, 40]. One study showed that the number of nodes found is associated with lymphocytic infiltration of the primary tumor, with more nodes being found in patients with prominent lymphocytic infiltration into the primary tumor than in patients with mild lymphocytic infiltration [41]. The differences in anatomical, physiological, and molecular characteristics between right-sided colon cancer and left-sided colon cancer may also influence the number of lymph nodes examined. [13] The benefits associated with high total number of lymph nodes might actually reflect the host lymphocytic reaction to tumor, which is associated with lymph node count [21, 41]. This phenomenon is commonly observed in lymph nodes draining the cancer. It has been postulated that the right colon mesentery contains a more complex lymphatic system, which leads to an enhanced immune response and thereby to increase in the number of lymph nodes examined in right-sided colon cancer, but there is no clear evidence in support of this theory [42]. A cadaveric study may help clarify this issue.

## Conclusion

In summary, the study demonstrated that it may not be correct to use the threshold of total number of lymph nodes ≥12 for predicting prognosis in both left-sided colon cancer and right-sided colon cancer in stage III patients with colon cancer. Although the number of lymph nodes evaluated is a prognostic factor, this does not mean that it is also a predictive factor [43]. Presently, many undersampled patients receive unnecessary treatment, and so adequate nodal evaluation is important. There is little evidence to justify adjuvant treatment in node-negative patients when too few nodes are evaluated. Based on the results of the present study, the surgeon should take tumor location into consideration when deciding on the minimum total number of lymph nodes to be retrieved.

## Additional files

Additional file 1: **Figure. S1.** (A) Survival curves for low- and high- total number of lymph nodes patients in the left-sided colon cancer cohort. (B) Survival curves stratified by lymph nodes ratio in the left-sided colon cancer cohort. (C) Survival of lymph nodes ratio 1 patients according to total number of lymph nodes in the left-sided colon cancer cohort. (D) Survival of lymph nodes ratio 2 patients according to total number of lymph nodes in the left-sided colon cancer cohort. (E) Survival of lymph nodes ratio 3 patients according to total number of lymph nodes in the left-sided colon cancer cohort. (F) survival of lymph nodes ratio 4 patients according to total number of lymph nodes in the left-sided colon cancer cohort. (PDF 1077 kb)

Additional file 2: **Figure S2.** (A) Survival curves of low- and high- total number of lymph nodes patients in the right-sided colon cancer cohort. (B) Survival curves stratified by lymph nodes ratio in the right-sided colon cancer cohort. (C) Survival of lymph nodes ratio 1 patients according to total number of lymph nodes in the right-sided colon cancer cohort. (D) Survival of lymph nodes ratio 2 patients according to total number of lymph nodes in the right-sided colon cancer cohort; (E) Survival of lymph nodes ratio 3 patients according to total number of lymph nodes in the right-sided colon cancer cohort. (F) Survival of lymph nodes ratio 4 patients according to total number of lymph nodes in the right-sided colon cancer cohort. (PDF 1068 kb)

## Abbreviation

SEER: Surveillance Epidemiology and End Results
