# Supplementary figures and images for: Prognostic value of total number of lymph nodes retrieved differs between left-sided colon cancer and right-sided colon cancer in stage III patients with colon cancer
Source: BMC Cancer. 2018 May 11;18:558. doi: 10.1186/s12885-018-4431-5 (PMC5948673; doi:10.1186/s12885-018-4431-5)

Figure S1

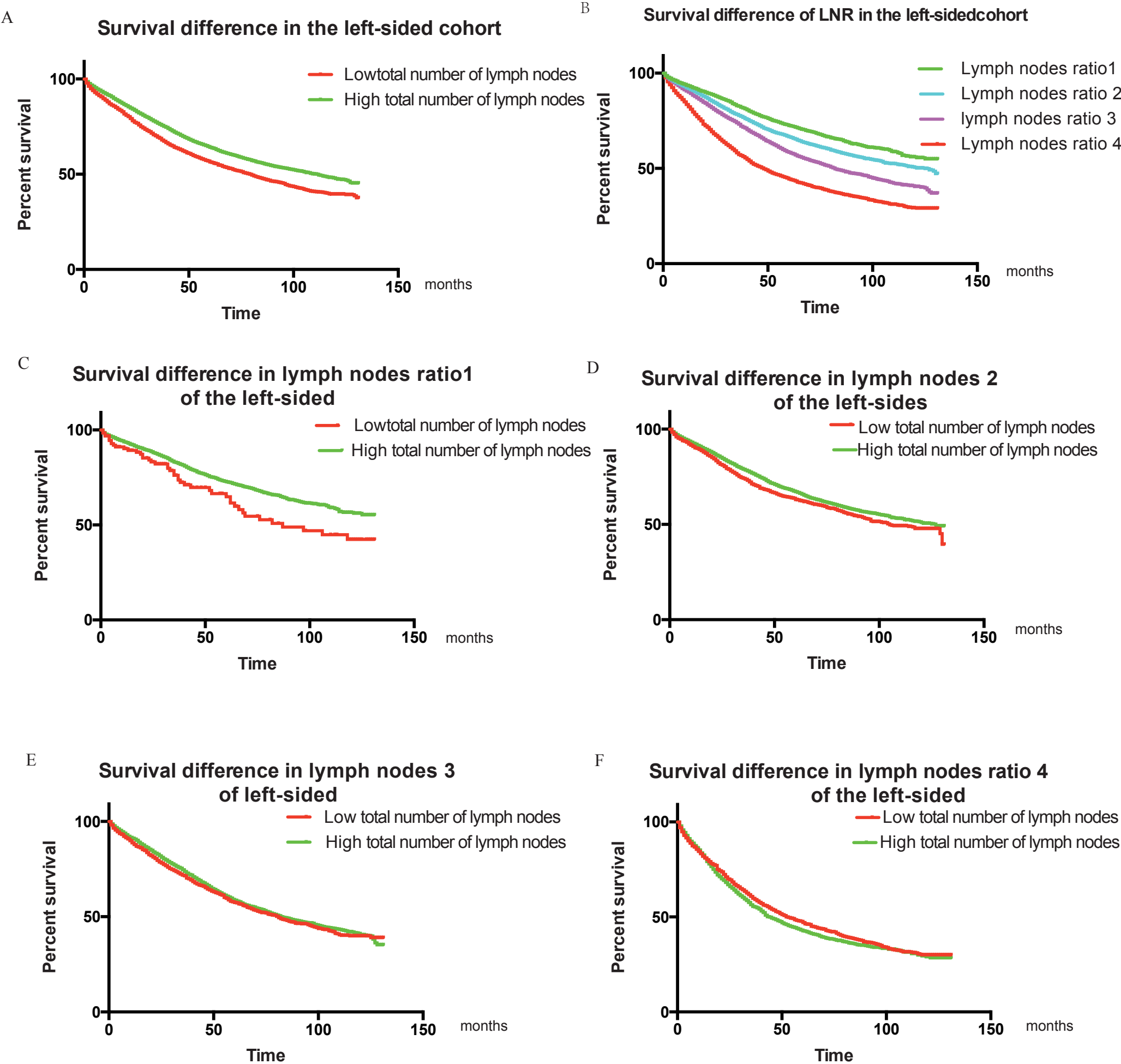

Supplement: Supplementary file 1 — Figure. S1. (A) Survival curves for low- and high- total number of lymph nodes patients in the left-sided colon cancer cohort. (B) Survival curves stratified by lymph nodes ratio in the left-sided colon cancer cohort. (C) Survival of lymph nodes ratio 1 patients according to total number of lymph nodes in the left-sided colon cancer cohort. (D) Survival of lymph nodes ratio 2 patients according to total number of lymph nodes in the left-sided colon cancer cohort. (E) Survival of lymph nodes ratio 3 patients according to total number of lymph nodes in the left-sided colon cancer cohort. (F) survival of lymph nodes ratio 4 patients according to total number of lymph nodes in the left-sided colon cancer cohort. (PDF 1077 kb) [file 12885_2018_4431_MOESM1_ESM.pdf]

Figure S2

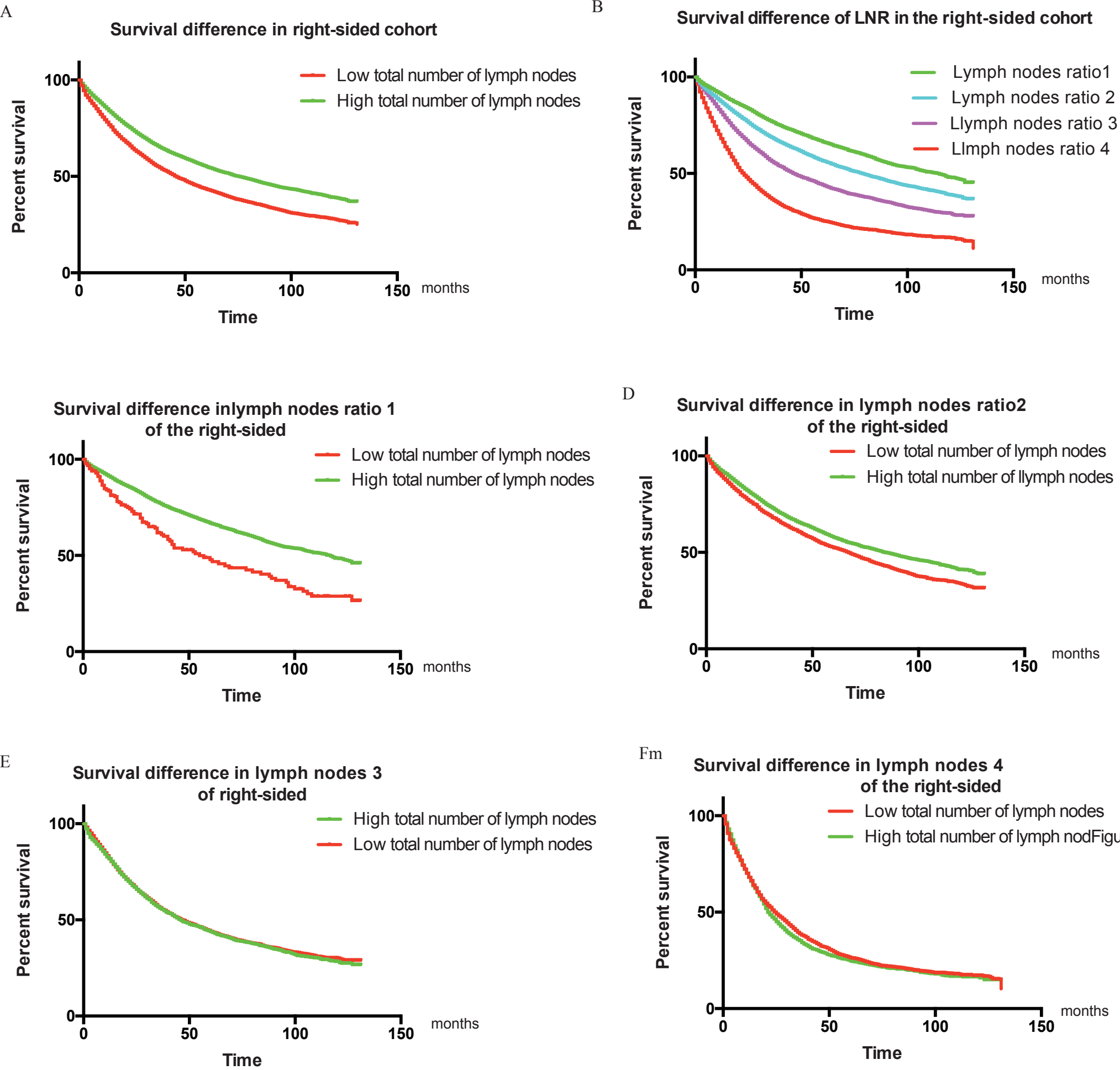

Supplement: Supplementary file 2 — Figure S2. (A) Survival curves of low- and high- total number of lymph nodes patients in the right-sided colon cancer cohort. (B) Survival curves stratified by lymph nodes ratio in the right-sided colon cancer cohort. (C) Survival of lymph nodes ratio 1 patients according to total number of lymph nodes in the right-sided colon cancer cohort. (D) Survival of lymph nodes ratio 2 patients according to total number of lymph nodes in the right-sided colon cancer cohort; (E) Survival of lymph nodes ratio 3 patients according to total number of lymph nodes in the right-sided colon cancer cohort. (F) Survival of lymph nodes ratio 4 patients according to total number of lymph nodes in the right-sided colon cancer cohort. (PDF 1068 kb) [file 12885_2018_4431_MOESM2_ESM.pdf]
